# MODERNIZING CHINA’S PRIMARY HEALTHCARE: REVISITING THE BAREFOOT DOCTOR PROGRAM TO ELEVATE SPECIALIZED NURSING

**DOI:** 10.1093/geroni/igae098.4383

**Published:** 2024-12-31

**Authors:** Qingwei Wang, Zixuan Wu

**Affiliations:** Xi’an Jiaotong-Liverpool University, Suzhou, Jiangsu, China; Johns Hopkins University, Baltimore, Maryland, United States

## Abstract

A persistent challenge in China’s primary healthcare system is the shortage of qualified healthcare professionals, a problem exacerbated by the decline of the Barefoot Doctor (BD) program. The newly innovative introduction of Specialized Nurses (SNs) in 2024 seeks to address physician shortages by equipping nurses with advanced skills, including prescription authority. This article explores how lessons from the BD program can inform the modernization of China’s primary care workforce with a focus on the needs of older adults. Through a comparative analysis, the study examines how both initiatives aim to address the shortage of healthcare professionals by harnessing grassroots power. Three critical themes are explored: 1) Community integration of Specialized Nurses (SNs) may face challenges due to prevailing cultural perceptions of nursing professionalism and the confinement of SNs to tertiary hospitals, which significantly hinder their involvement in community healthcare; 2) The effectiveness of the program is constrained by insufficient financial support and unclear career progression paths; 3) Sustainable caregiving strategies are necessary that address both traditional and contemporary health needs, expanding beyond the BD’s focus on environmental and infectious diseases to include non-communicable diseases. Drawing from the BD model, future efforts should prioritize community-based care, expanding opportunities for home visits, especially for older adults who may face mobility challenges.. Ensuring adequate fiscal funds and active agency involvement from the government and health administrators is crucial for sustaining the primary healthcare system. Additionally, preventive care should adopt a holistic and interdisciplinary approach that integrates diet, exercise, and psychological care.

